# Lipid Status of A2780 Ovarian Cancer Cells after Treatment with Ruthenium Complex Modified with Carbon Dot Nanocarriers: A Multimodal SR-FTIR Spectroscopy and MALDI TOF Mass Spectrometry Study

**DOI:** 10.3390/cancers14051182

**Published:** 2022-02-24

**Authors:** Maja D. Nešić, Tanja Dučić, Manuel Algarra, Iva Popović, Milutin Stepić, Mara Gonçalves, Marijana Petković

**Affiliations:** 1 Center for Light-Based Research and Technologies COHERENCE, Department of Atomic Physics, Vinča Institute of Nuclear Sciences, National Institute of the Republic of Serbia, University of Belgrade, 11000 Belgrade, Serbia; ivavukicevic@vin.bg.ac.rs (I.P.); mstepic@vin.bg.ac.rs (M.S.); marijanapetkovic@vin.bg.ac.rs (M.P.); 2 ALBA-CELLS Synchrotron, MIRAS Beamline, 08290 Cerdanyola del Vallès, Spain; tducic@cells.es; 3 INAMAT2—Institute for Advanced Materials and Mathematics, Department of Science, Public University of Navarre, Campus de Arrosadia, 31006 Pamplona, Spain; 4 CQM—Centro de Química da Madeira, Universidade da Madeira, 9020-105 Funchal, Portugal; maraisabel@staff.uma.pt

**Keywords:** anticancer Ru metallodrug, carbon dots, N-doped carbon dots, drug nanocarriers, lipids, MALDI TOF MS, SR-FTIR spectroscopy

## Abstract

**Simple Summary:**

Developing new anticancer medicaments is focused on inducing controlled elimination of tumor tissue without severe side effects. It is essential to enable the medicament to reach the target molecule without provoking the immune response too early. The first cellular changes might occur already at the level of the cell membrane, composed mainly of lipids. Therefore, we used spectroscopic techniques to study the interaction of potential metallodrug [Ru(η^5^-C_5_H_5_)(PPh_3_)_2_CN] (RuCN) with lipids of A2780 ovarian cancer cells and investigated if these changes are affected by the presence of drug carriers (carbon dots (CDs) and nitrogen-doped carbon dots (N-CDs)). Our results showed that CDs and N-CDs prevent lysis and moderate oxidative stress of lipids caused by metallodrug, still keeping the antitumor activity and potential to penetrate through the lipid bilayer. Therefore, Ru drug loading to carriers balances the anticancer efficiency and leads to better anticancer outcomes by reducing the oxidative stress that has been linked to cancer progression.

**Abstract:**

In the last decade, targeting membrane lipids in cancer cells has been a promising approach that deserves attention in the field of anticancer drug development. To get a comprehensive understanding of the effect of the drug [Ru(η^5^-Cp)(PPh_3_)_2_CN] (RuCN) on cell lipidic components, we combine complementary analytical approaches, matrix-assisted laser desorption and ionization time-of-flight mass spectrometry (MALDI TOF MS) and synchrotron radiation-based Fourier transform infrared (SR-FTIR) spectroscopy. Techniques are used for screening the effect of potential metallodrug, RuCN, without and with drug carriers (carbon dots (CDs) and nitrogen-doped carbon dots (N-CDs)) on the lipids of the human ovarian cancer cell line A2780. MALDI TOF MS results revealed that the lysis of ovarian cancer membrane lipids is promoted by RuCN and not by drug carriers (CDs and N-CDs). Furthermore, SR-FTIR results strongly suggested that the phospholipids of cancer cells undergo oxidative stress after the treatment with RuCN that was accompanied by the disordering of the fatty acid chains. On the other hand, using (N-)CDs as RuCN nanocarriers prevented the oxidative stress caused by RuCN but did not prevent the disordering of the fatty acid chain packing. Finally, we demonstrated that RuCN and RuCN/(N-)CDs alter the hydration of the membrane surface in the membrane–water interface region.

## 1. Introduction

The cell membrane is a complex and highly diverse cellular component, mainly composed of a large variety of different lipids [1]. Any drug must interact first with the membrane to cross it and reach its intracellular target(s). For this reason, the efficiency of drugs’ interaction with the constituents of the membrane, primarily membrane lipids, is one of the pharmacological features playing a crucial role in their biological activity [2]. Membrane domains act as platforms for a whole range of cellular functions, such as cell proliferation, adhesion, migration, and intracellular trafficking. Moreover, fatty acids and related phospholipids are well-known signaling molecules with pro- and anti-inflammatory functions [3]. Therefore, the role of membrane lipids in regulating numerous cellular functions has led to the emergence of cell lipids as an alternative molecular drug target. On the other hand, as a cell’s barrier, the cell membrane plays an important role in the drug’s absorption, metabolism, and excretion. The interaction of drug molecules with the lipid membrane leads to changes in the physicochemical properties, pharmacological activity, and bioavailability of the drug. This interaction could also lead to changes in lipid structure and membrane integrity [4].

The Ru complexes with finely tuned ligands can create diverse modes of interaction with biomolecular targets, thus providing a high potential for the development of anticancer drugs. These complexes have shown superior anticancer profiles such as increased selectivity toward cancer cells, inhibition of tumor growth or metastasis, lower drug resistance, and less toxicity against healthy cells [5]. Currently, several Ru complexes are under clinical trials for the treatment of solid tumors, such as non-small-cell lung cancer, colorectal carcinoma, gastrointestinal neuroendocrine tumors, and bladder cancer [5]. In addition, the combinational use of Ru complexes with existing clinical antitumor drugs is under study [6,7]. Further optimization of Ru metallodrugs’ structure with relevant modification is a good strategy to improve their targeting capability and antitumor activity. The use of drug carriers is an effective strategy that could alter the anticancer drug mechanism of action by triggering different signaling pathways compared to the drug itself. The nanoscale size of the nanoparticles allows their selective accumulation in the cancer tissue by the enhanced permeability and retention (EPR) effect [8]. Furthermore, the coordination of anticancer drugs to nanocarriers has been shown to improve the chemotherapeutic action of drugs in terms of better drug solubility, enhanced cellular uptake, efficiency, selectivity, and reduction in toxicity [9,10].

In this work, we use a Ru complex, [Ru(η^5^-C_5_H_5_)(PPh_3_)_2_CN], as the potential anticancer agent because it was shown previously that organometallic Ru compounds with η5-cyclopentadienyl ligand exhibited attractive pharmacological properties for cancer therapy [11,12,13]. The aromatic ligand present in the structure of the complex allows the stability and protection of the metal Ru(II) center. As a drug nanocarrier, we used carbon dots (CDs) and N-doped carbon dots (N-CDs). The surface of CDs can be easily functionalized due to sp^2^/sp^3^ hybrid structure that can load aromatic drugs via strong π–π interactions. Furthermore, these nanoparticles have high water dispersity, nontoxicity, and good biocompatibility, which have already led to significant interest in CDs for nano-based drug carrier applications [14,15,16]. In addition, N-CDs have –NH_2_ functional groups that are sufficiently soluble in water and could interact with various drug functional groups and biomolecules, which is essential for drug loading and thus a drug carrier [17,18]. In this study, we investigate the influence of RuCN on the membrane lipid structure and function, which is often omitted in drug-related studies, and assess the effects of (N-)CDs as nanocarriers.

Ovarian cancer A2780 cell line is used as a model system that represents endometrioid carcinomas, a special pathological type of epithelial ovarian cancer. In developed countries, epithelial ovarian cancer is one of the most common cancers and the most lethal gynecological malignancy because most of the cases are reported with advanced disease due to the absence of specific biomarkers and the asymptomatic characteristics of the disease at early stages [19]. Despite the fact that this kind of ovarian cancer is not categorized as high-grade serous, it is linked to endometriosis, a disease that affects 10% of the female population [20]. At present, standard platinum–taxane chemotherapy remains among the main treatment strategies along with surgery. In the advanced stages, patients show poor rates of response to chemotherapy and most patients ultimately develop recurrent or progressive disease. Moreover, there has only been one major new treatment introduced in ovarian cancer treatment in the last 30 years [21]. Better understanding the consequences of drug–lipid membrane interaction could provide promising novel treatment approaches and help overcome the current gap in the translation of laboratory research to novel therapies for ovarian cancer patients.

## 2. Materials and Methods

### 2.1. Materials

CDs and N-CDs were prepared and characterized as previously described [22,23]. Organoruthenium complex, cyano(cyclopentadienyl)bis(triphenylphosphine)ruthenium(II), [Ru(η^5^-Cp)(PPh_3_)_2_CN] (RuCN), was prepared by following a published method [24].

A2780 ovarian cancer cell line was obtained from the European Collection of Cell Cultures (ECACC, Salisbury, UK). The cell culture medium, fetal bovine serum (FBS), and antibiotics were purchased from Life Technologies (Paisley, UK). Resazurin sodium salt (75.0% of dye content) was bought from Sigma Aldrich and dissolved in PBS (phosphate buffer solution, Sigma Aldrich) to prepare the solution (0.1 mg/mL) for the cytotoxicity assays.

Matrix for MALDI TOF MS (2,5-dihydroxybenzoic acid (DHB), octan-1-ol, methanol, and trifluoroacetic acid (TFA)) was purchased from Sigma-Aldrich (Taufkirchen, Germany) and used without further purification.

### 2.2. Determination of the Cytotoxicity of RuCN, CDs, N-CDs, and RuCN/(N-)CDs

A2780 cells were grown in RPMI-1640 medium containing 10% FBS and 1% antibiotic–antimycotic solution. Afterward, cells were harvested and seeded in 96-well plates at a density of 10,000 cells/well and incubated with RuCN (75 µM), (N-)CDs (50 µg/mL), and their combinations (RuCN/(N-)CDs) at the same final concentrations. The concentration of RuCN was determined from preliminary experiments (Figure S1). The cytotoxicity was evaluated after 48 h exposure using the resazurin assay, in which 200 µL of fresh supplemented RPMI-1640 medium with 0.01 mg/mL resazurin was added to each well and incubated for another 4 h. Afterward, the relative resorufin fluorescence was measured (λex = 530 nm, λem = 590 nm) using a Victor 3 1420 Perkin Elmer microplate reader (Waltham, MA, USA). Four replicates were conducted for each treatment (*n* = 4). Nontreated cells were used as controls. Results were presented as a percentage of control (100% metabolic activity).

### 2.3. Determination of the Lipophilicity of (N-)CDs

Lipophilicity of the nanocarriers, CDs and N-CDs, was determined as the log value of partition coefficients of nanocarriers between water and octan-1-ol [25]. The amount of 2 μL of each nanocarrier solution was applied on two thin-layer chromatography (TLC) plates (silica gel 60, Merck, Darmstadt, Germany). After drying at room temperature, each plate was placed in a developing tank with 5 mL of octan-1-ol or 5 mL of distilled water. When solvents reached 1 cm from the top of the TLC plates, plates were removed and left to dry at room temperature. The visualization was performed by placing the plates in the wide glass pots with crystalline I_2_. The R_f_ values were determined for each nanocarrier in octan-1-ol and water. The lipophilicity, log of the partition coefficient (log K_o/w_), was calculated from the following equation:

log K_o/w_= logRf_o_/Rf_w_ (1)

Rf_o_ is a ratio of the migration distance, given in cm, traveled by nanocarrier and the solvent front (octan-1-ol), while Rf_w_ is the ratio of distance traveled by nanocarrier and the solvent front (water).

### 2.4. Extraction of Cellular Lipids for MALDI TOF MS Analysis

Cells were prepared and treated the same way as described for the cytotoxicity experiment. After they were incubated with RuCN with and without (N-)CDs for 24 h, total lipid extraction from A2780 cells was obtained according to the protocol of Bligh and Dyer [26]. Briefly, four steps were carried out: (i) addition of 750 μLof chloroform/methanol mixture (1:2, *v*/*v*) to the cell pellet and vortexing the resulting solution, (ii) addition of 16.8 μL of hydrochloric acid (6 M) and 250 μL of chloroform and vortexing of the solution, (iii) addition of 250 μL of desalted water and vortexing of solution again, (iv) incubating the resulting solution at 4 °C for1 h and centrifugation for 5 min at 300× *g* to achieve the separation between the two phases. The total lipid extracts were collected in the lower phase.

### 2.5. Matrix-Assisted Laser Desorption/Ionization Mass Spectrometry (MALDI TOF MS)

DHB matrix (0.5 M in MeOH, 0.1% TFA) was used as the MALDI matrix to analyze lipid extracts from A2780. Premix of the samples and the matrix was applied as 1.5 μL droplets on the sample plate and left at room temperature to cocrystallize. The MALDI TOF MS measurements were performed with an AutoflexmaX mass spectrometer (Bruker Daltonics, Bremen, Germany) equipped with a 355 nm N_2_ laser with the maximum repetition rate of 2 kHz. All mass spectra were acquired in the positive reflector mode with delayed extraction. Spectra were calibrated by setting the peak of the protonated DHB matrix to its appropriate value (155.034 Da).

### 2.6. Preparation of the Cells for Synchrotron Radiation Fourier Transform Infrared (SR-FTIR) Spectroscopy

Cells were grown to subconfluence and then were seeded at 60,000 cells/well in 24-well plates on a round 10 × 0.5 mm^2^ CaF_2_ carrier and treated with RuCN, (N-)CDs, and RuCN/(N-)CDs as described in the cytotoxicity experiment. After the treatment, plates were incubated at 37 °C in a humidified atmosphere of 5% CO_2_ for 24 h. Afterwards, they were washed with PBS twice and lyophilized. Lyophilization was carried out using a Martin Christ Alpha1–2 freeze-dryer lyophilizer and Labconco Freeze Zone 4.5 LiterFreeze Drier Systems (Osterode am Harz, Germany) at the temperature of −50 °C and the vacuum of 0.3 mBa for 24 h.

### 2.7. SR-FTIR Spectroscopy

After the treatment, changes in lipid membrane biomolecules were analyzed by using SR-FTIR spectroscopy (Synchrotron ALBA, MIRAS beamline, Barcelona, Spain). Synchrotron light was used as an IR source, and a 3000 Hyperion microscope was coupled to a Vertex 70v spectrometer; mercury cadmium telluride (MCT) cooled with liquid nitrogen was used as a detector. The aperture of the FTIR microscope was set to a size of a single cell (12 × 12 μm^2^), and 60 cells were analyzed from each group, taking into account the lipid signature. Three independent replicates were conducted by co-adding 256 spectra at 4 cm^−1^ resolution. The spectroscopic data were collected in transmission mode using the 36× Schwarzschild objective and condenser. Spectra for each treatment were collected in the 4000–700 cm^−1^ mid-infrared range. The OPUS 8.2 (Bruker, Germany) software package was used for data acquisition.

### 2.8. Data Processing and Statistical Evaluation

SR-FTIR spectra were processed using the Quasar Spectroscopy tools [27], which allows multimodal spectral analysis involving the principal component analysis (PCA).

MALDI TOF mass spectra were processed using the instrument software, Flex analysis 2.2 (Bruker Daltonics, Bremen, Germany), which was used to determine the peak S/N values.

Statistical differences between treatments were obtained using one-way analysis of variance (ANOVA) with Tukey’s multicomparison post-test using GraphPad Prism 8.0.1. In all cases, *p*-value < 0.05 was considered significant.

Deconvolution of C=O band (1760–1725 cm^−1^) was completed by OriginPro (version 2021, 21-day trial version) using Gaussian functions to obtain the best fit for the band shapes of the experimental carbonyl vibrations in recorded spectra. In all cases, two underlined bands at 1742 and 1735 cm^−1^ were used to simulate the experimental line shapes.

## 3. Results and Discussion

Despite the evident importance of studying changes in cell lipid structure caused by drugs and their essential functions, they have not been studied as thoroughly as other biological molecules, such as proteins, nucleic acids, or carbohydrates. Since the natural membrane is a very complex system, lipid analysis imposes severe technical challenges. Therefore, as useful alternatives, isolated biomolecules and model membrane systems are used most commonly to investigate the effects of drug molecules on the membrane structure. To understand drug–membrane interaction phenomena, we employed complementary analytical techniques, MALDI TOF MS (Funchal, Portugal) and FTIR spectroscopy (Barcelona, Spain), to analyze potential Ru-metallodrug and drug-carrier effects on the lipid membrane of the human ovarian cancer line A2780. This type of cancer is selected for investigation in view of the fact that it is the most common cause of death among gynecologic malignancies in developed countries [28] and has a natural tendency to relapse and the ability to develop resistance to traditional therapies [29,30]. While the MALDI TOF MS was used to track the effects of drug molecules on the isolated lipids and identify them, FTIR spectroscopy tracked specific chemical events after the treatments in the lipid region of all individual living cancer cells. This dual approach is helpful because the effect of any potential therapeutic on isolated biomolecules could differ from the effect of the same system embedded in the intracellular milieu.

### 3.1. Evaluation of Anticancer and Physicochemical Properties of the Ru Complex and Drug Carriers (CDs and N-CDs)

**Figure 1:**
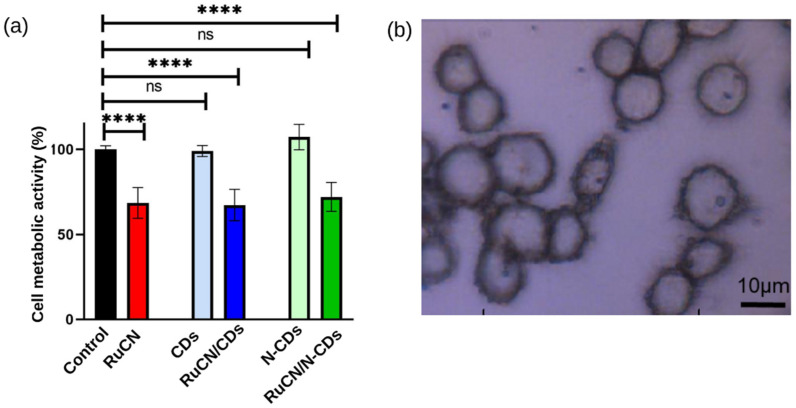
(**a**) Cell metabolic activity after the indicated treatments is expressed as a percentage of the nontreated cells (control) (the mean ± SD). Statistical significance was determined using the ANOVA test. ns, not significant, *p* > 0.05; ****, significant, *p* < 0.0001. (**b**) A microscopic image shows the original morphology of treated A2780 cancer cells. The microscopic images of control and cells with all treatment conditions are presented in Figure S2.

The effects of treatments with potential metallodrugs with and without nanocarriers on the A2780 cancer cells are presented in Figure 1. The survival rate of cells decreased to 67%compared to the control after RuCN treatment without and with both nanocarriers (Figure 1a). The addition of nanocarriers does not further decrease cell viability, indicating that potential anticancer activity originates from the Ru complex. Besides, the cytotoxicity caused by (N-)CDs alone does not significantly differ from the control/untreated cells.

Cell death is a complex event involving phospholipids and signaling proteins, and the change of membrane dynamics may be, in fact, the result of a change of biomolecular networks in the membrane. Interaction studies with a particular focus on the cell lipid components cannot be overlooked during the design and synthesis of a drug because lipids might be the main targets of its action. The drug’s ability to interact with membrane lipids and cross the membrane largely depends on the physicochemical properties of both the membrane and the drug. Therefore, knowing the physicochemical properties of the drug is important to predict and understand the transport and impact of drugs on the cell membrane lipids. The uptake of NPs by cells can be considered as a two-step process consisting of binding the NPs to the cell membrane and internalization. The first process appears to be most affected by the surface charge, size, and lipophilicity of the NPs; we examined these properties for RuCN, CDs, and N-CDs used in our study, and the results are summarized in Table 1. We aimed in this study to correlate their physicochemical properties with cytotoxicity and effect on the membrane lipids of cancer cells.

Ru complex in physiological conditions, pH ~7, is neutral and micrometer-sized and has a positive value for lipophilicity (Table 1). The complex is hydrophobic due to the lipophilic nature of PPh_3_ and Cp ligands. Considering the cell membrane’s hydrophobic nature, small, neutral, and hydrophobic molecules, such as used complex, should pass through the membrane more easily than hydrophilic, large ones. On the other hand, highly hydrophobic drugs may remain trapped in the lipid membrane by strong hydrophobic bonding and destroy the membrane integrity [2].

Studied drug carriers did not differ in their mean size, 7–8 nm (Table 1). Still, they have differences in electrical charge: while N-CDs have a positive charge, CDs have a more negative charge, which is consistent with functional groups on the surface. The positive charge on N-CDs originates from protonated amine groups, whereas the negative charge on CDs arises from the –COO^−^ groups. Since the cell membrane is negatively charged, in particular in the inner leaflet, due to the presence of phosphatidylserine, positively charged nanoparticles interact favorably with the cell membrane via electrostatic interactions and are more internalized by cells than neutral or negatively charged nanoparticles [31,32]. According to that, we could expect stronger interactions between N-CDs and membrane lipids than with CDs. However, there has been evidence of cellular uptake of negatively charged particles [33,34,35], suggesting that electrostatic interactions contribute to nanoparticle–cell interactions to some degree, but a complex contribution from several factors must be taken into account. The obtained negative values for nanoparticle lipophilicity (Table 1) mean the nanoparticles have a higher affinity for the aqueous phase;i.e., they are hydrophilic. Still, N-CDs showed a more hydrophilic nature than CDs, implying that CDs could pass through the membrane more easily than N-CDs. Considering the combined contribution of the physicochemical properties of the complex and nanocarriers, we checked their contribution to changes in cell lipid structure.

### 3.2. MALDI TOF MS Analysis

**Figure 2:**
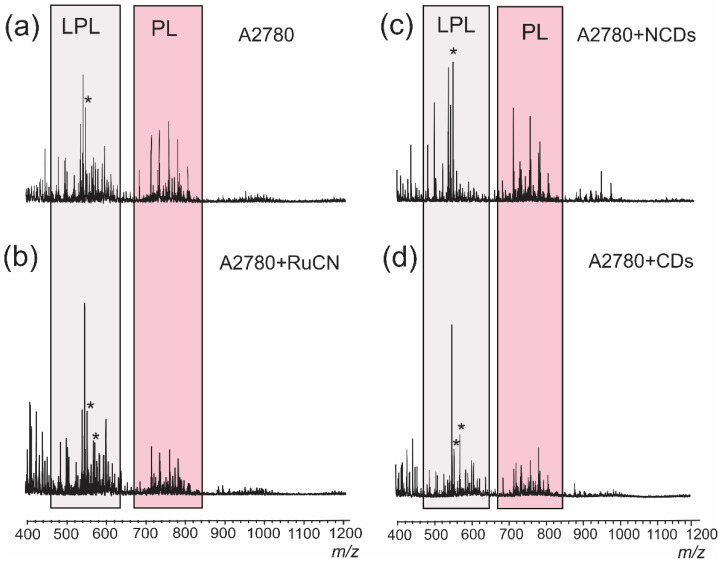
Positive-ion MALDI TOF mass spectra of extracted lipids from the A2780 cancer cell line (**a**) after the treatment with Ru-CN metallodrug (**b**), N-CDs (**c**), and CDs (**d**). Peaks with an asterisk (*) correspond to the higher molecular weight oligomers of the DHB matrix. For the assignment, see Table 2.

MALDI results gave insights into the lipid composition of the human ovarian cancer cell line (Table 2, Figure 2). They also provided information about the possible changes in overall lipid composition and lipid degradation after the treatment with metallodrug and carriers.

The major lipids identified by MALDI TOF MS belonged to multiple subclasses, including phosphatidylcholine (PC), lysophosphatidylcholine (LPC), phosphatidylethanolamine (PE), lysophosphatidylethanolamine (LPE), phosphatidylserine (PS), lysophosphatidylserine (LPS), phosphatidylinositol (PI), phosphatidic acid (PA), lysophosphatidic acid (LPA), and phosphatidylglycerol (PG). All signals detectable in the MALDI TOF mass spectra are divided into two regions (460–620 and 670–840 *m*/*z*) which were marked as lysophospholipids (LPs) and phospholipids (PLs), respectively (Figure 2). The region at *m*/*z* > 850 contains signals from triacylglycerols, phosphatidylinositols (PIs), and/or phosphatidylserines (Table 2), which were not analyzed. Although PIs are relevant for signal transduction, this PL species is hardly detectable in the MALDI mass spectra of lipid mixtures [36]. Therefore, for the assessment of the level of PL degradation between different treatments (RuCN, CDs, N-CDs), we analyzed the extent of LP production and monitored the ratio between SN (signal-to-noise) ratio values of PLs and LPs.

**Figure 3:**
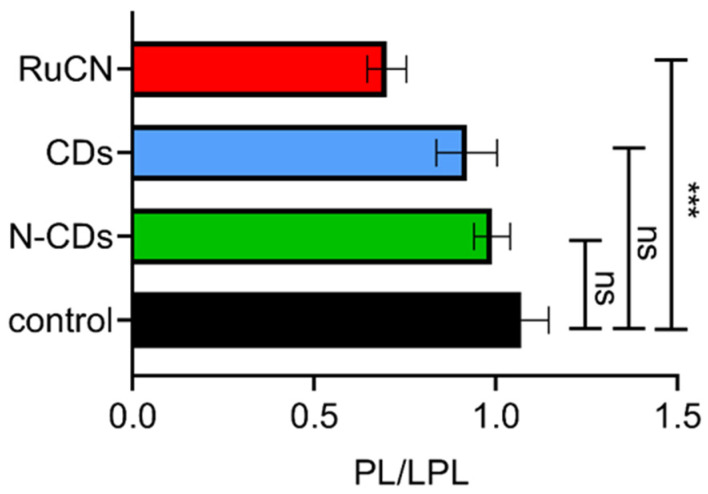
The ratio between SN values of PLs and LPs (PL/LPL) was obtained from MALDI TOF spectra after the treatment with Ru-CN metallodrug, N-CDs, and CDs. All the values are compared to the control (PL and LP ratio of untreated cells). ns, not significant, *p* > 0.05; ***, significant, *p* ≤ 0.001.

Obtained results revealed significantly higher content of lyso-lipids when cancer cells are treated with Ru metallodrug (Figure 3) compared to untreated cancer cells (control). On the contrary, incubation of cancer cells with carriers (CD and N-CDs) did not produce an elevated level of lyso-lipids compared to control. Obtained results reveal that RuCN promoted hydrolysis of membrane lipids and are consistent with cytotoxicity (Figure 1). On the other hand, enhanced PL hydrolysis can be a consequence of an increased level of oxidative stress, as demonstrated by in vitro experiments with commercially available PLs [37]. Moreover, previous works have established that some classes of drugs linked with phospholipidosis can promote lipid hydrolysis in model systems in vitro [38,39]. Such membrane-active drugs affect the rate of lipid hydrolysis by either changing the bulk properties of the membrane, mediated by secondary effects on interfacial water activity, or by direct involvement in acid or base catalysis. Drug selectivity for lipidation and hydrolysis is complex and linked to several factors, including the molecular disposition in the membrane interface, drug distribution profile (log D), and the pKa values of any ionizable groups [40]. However, hydrophobic interactions between acyl chains of membrane lipids and aromatic PPh_3_ and Cp nonpolar RuCN ligands can enhance the further reactions between the membrane and RuCN. Furthermore, lipid modulation induced by RuCN could trigger signaling pathways that could be responsible for the antitumor activity of this potential metallodrug.

### 3.3. SR-FTIR Spectroscopy Analysis

#### 3.3.1. Oxidative Stress Checking after the Treatments

**Figure 4:**
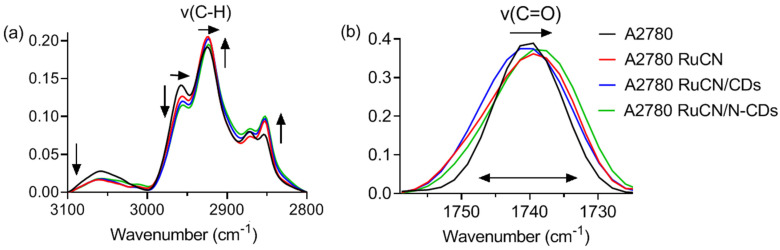
The lipid fingerprint area ((ν(C–H) (**a**) and ν(C=O) (**b**)) of the untreated A2780 cancer cells (black line) and cells treated with RuCN complex (red line), RuCN/N-CDs (green line), and RuCN/CDs (blue line).

SR-FTIR spectroscopy provides a unique signature and detailed information about the structure and organization of cell lipids in the membrane [28,29]. Therefore, the spectral variations in specific spectral signatures after the cellular treatment can be related to anticancer drugs’ mechanism of action with and without drug carriers. A2780 cancer cell line was exposed to RuCN and RuCN/(N-)CDs for 24 h. FTIR lipid fingerprint spectral region of cancer cells with and without the treatment was recorded and is presented in Figure 4 for each condition.

We selected two intervals that are crucial for lipid analysis (Figure 4). The higher wavenumber interval of the spectrum from 3100 to 2800 cm^−1^ is related to contribution from carbon–hydrogen stretching vibrations (ν(C–H)), which originates from the fatty acid chains of lipids (Figure 4 a). At the same time, the lower wavenumber region of the spectrum (between 1780 and 1690 cm^−1^) corresponds to the stretching of the carbonyl groups (ν(C=O)) (Figure 4b). A description of the assigned absorption bands is listed in Table 3. The contribution from phosphate groups at 1240 cm^−1^ (ν_as_(PO_2_^−^)) and 1090 cm^−1^ (ν_s_(PO_2_^−^)) was also detected in the spectrum. However, as these groups are constituents mostly of nucleic acids, IR bands originating from phosphate group stretching vibrations were excluded from the analysis.

In the higher wavenumber interval (Figure 4a), the most pronounced absorption bands are localized at 2960 and 2925 cm^−1^, which correspond to the asymmetric vibration of the methyl (ν_as_(CH_3_)) and methylene groups (ν_as_(CH_2_)), respectively. The bands that correspond to the symmetric vibration of methyl (ν_s_(CH_3_)) and methylene groups (ν_s_(CH_2_)) are localized at 2875 and 2850 cm^−1^, respectively. The spectral behavior of the bands in the lipid region is very characteristic of lipid oxidation. The apparent intensity variation in the spectral data after the cell treatment compared to control (given by black line color) represents the change in absorbance, which is proportional to the concentration of the methyl, methylene, and ethylene groups. Thus, the intensity variation observed in the lipid fingerprint area can be attributed to the peroxidation process of lipids induced by the treatment of cells with RuCN or RuCN/(N-)CDs. The most apparent changes in the lipid fingerprint region were an increase in the intensity of ν_as_(CH_2_) and ν_s_(CH_2_) and a decrease in the intensity of νCH(C=C–H) and ν_as_(CH_3_) in cells after the treatments compared to control (Figure 4a). The intensity of the νCH(C=C–H) band at 3060 cm^−1^ correlates positively to the degree of unsaturation in fatty acids. A significant decrease in the νCH(C=C–H) band indicates a reduction in the concentration of unsaturated bonds, possibly caused by a peroxidation process.

**Figure 5:**
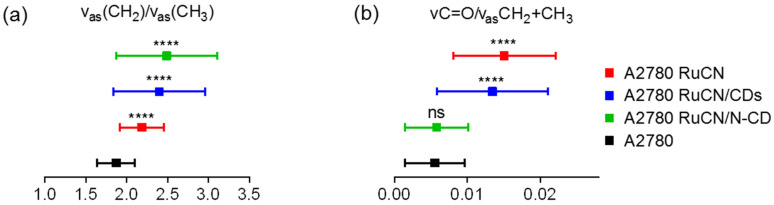
Analysis of oxidative stress markers. (**a**) Distribution of the ratios between asymmetric CH_2_ and CH_3_ bands (ν_as_(CH_2_)/ν_as_(CH_3_)). (**b**) Distribution of the ratio between the C=O band and the sum of asymmetric CH_3_ and CH_2_ bands (ν(C=O)/ν_as_(CH_2_ + CH_3_)). Values are presented with the mean ± SD. All the values are compared to the control (A2780 untreated cells, black color). **** indicates values that are significantly different (*p* ≤ 0.0001); ns, not significant (*p* > 0.05).

The most frequent parameters for oxidative stress are the ratio of the asymmetric vibration of both CH_2_ and CH_3_ (ν_as_(CH_2_)/ν_as_(CH_3_)) and the ratio of the carbonyl group and asymmetric vibration of both CH_2_ and CH_3_ (ν(C=O)/ν_as_(CH_2_+CH_3_)) [41]. Firstly, ν_as_(CH_2_)/ν_as_(CH_3_) may be considered as an indicator of fatty acid chain length and/or degree of saturation. The ratios of ν_as_(CH_2_)/ν_as_(CH_3_) significantly increased after all treatments, revealing that fatty acid chains became more saturated than in the control (Figure 5a).

To clarify whether these changes are caused by lipid peroxidation, we normalized the carbonyl group absorption to the total lipid content; i.e., the ratio of the C=O absorbance and the sum of all CH_2_ and CH_3_ absorbance was calculated. The ester carbonyl stretching vibration ν(C=O) band exhibits a maximum near 1740 cm^−1^, which originates from ester bonds between fatty acids and glycerol within the lipid. This type of bond may also be formed by the peroxidation of fatty acid chains as shorter lipid molecules such as malondialdehyde (MDA) and 4-hydroxynonenal (HNE), major end-products of oxidation of polyunsaturated fatty acids, both having C=O group [42]. Therefore an increase in the peak area of this band (Figure 4b) indicates a generation of primary oxidation products that point to an increase in lipid peroxidation in the cancer cells after the treatment. The ν(C=O)/ν_as_(CH_2_ + CH_3_) ratios before and after the treatments are presented in Figure 5b, and they were compared to the control. According to the results, the statistically significant increase in ν(C=O)/ν_as_(CH_2_ + CH_3_) values were detected after treatments with RuCN and RuCN/CDs. The results presented in Figure 5b strongly suggest that the lipids of the cancer cells undergo free radical attacks during oxidative stress mediated by RuCN and RuCN/CDs, while the interaction of Ru metallodrug with N-CDs leads to the obstruction of the Ru-metallodrug peroxidation ability.

The oxidative stress capability of RuCN positively correlates with its high lipophilicity. The carriers, which are more hydrophilic, lower the lipophilic properties of the drug by combining with it and blocking the functional groups that lead to the oxidative stress capability of the RuCN drug. According to literature sources, lipophilicity has been shown to be an important predictor of oxidative stress capability [43]. Highly lipophilic substances possess a higher membrane diffusion capacity and can easily be accumulated in the inner mitochondrial membrane, which leads to ROS formation and deterioration of the functional integrity of the respiration chain. The mechanism by which some anticancer drugs can regulate oxidative stress is through the promotion of cytochrome C from the mitochondria, while other drugs based on transition metals such as cisplatin and carboplatin generate very high ROS levels [44]. Moreover, some Ru complexes can increase the reactive oxygen species (ROS) generation in tumor cells, causing oxidative stress [45,46], and it is well known that transition metals may play a significant role in lipid peroxidation by catalyzing the decomposition of hydroperoxides [47,48]. The primary products of lipid peroxidation are lipid hydroperoxides (LOOH) which can be decomposed to oxygen radical intermediates such as lipid alkoxyl (LO•) and/or peroxyl radical (LOO•) in the presence of transition metal. This reaction results in lipid hydroperoxide decomposition and the oxidation or reduction of the metal [47]. For the Ru^2+^ metal ion, this could be represented by the following reactions:

LOOH + Ru^2+^→ LO• + OH^−^ + Ru^3+^ (2)

LOOH + Ru^3+^→ LOO• + H^+^ + Ru^2+^ (3)

The lipid LO• and LOO• radicals can attack other lipids, promoting the propagation of lipid peroxidation. Moreover, they can affect cell life or death through complex signaling cascades related to the fatty acid structure and their oxidation products [48]. Potential changes in the cell membrane caused by treatments, such as a decrease in the degree of fatty acid chain unsaturation or alternation in their packing within the membrane, can be attributed to the ROS attacks during oxidative stress [49,50,51]. This eventually leads to loss of membrane integrity, signaling processes, and metabolic processes. Therefore, we checked the effect of lipid peroxidation on the structural and dynamic properties of the membrane using the SR-FTIR spectroscopy approach.

#### 3.3.2. Impact of RuCN and RuCN/(N)CDs on the Structural and Dynamic Properties of the Cancer Cell Lipids

**Figure 6:**
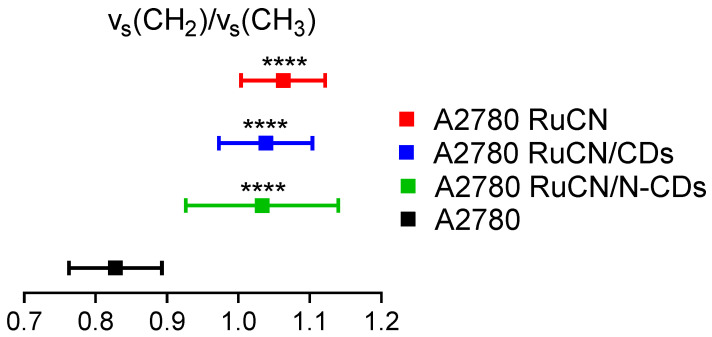
The ratios between symmetric CH_2_ and CH_3_ bands (ν_s_(CH_2_)/ν_s_(CH_3_)). The values are presented with the mean ± SD. All the values are compared to the control (A2780 untreated cells, black color), and **** indicates values that are significantly different (*p* ≤ 0.0001).

The frequencies of C–H stretching modes from the fatty acid chains are analyzed to gain information concerning the freedom of motion, fatty acyl order, acyl chain packing, and membrane fluidity in cancer cell membrane lipids caused by treatment. After the treatment with RuCN and RuCN/(N-)CDs, the shift of the ν_as_(CH_2_,CH_3_) frequency towards lower values was observed compared to control (Figure 4a), pointing toward an increased conformational order of the acyl chains in the membrane and a decrease in the cancer cell membrane fluidity [50,51,52]. The lower frequency can be linked with the higher saturation degree, increasing conformational order, and lower chain flexibility. Acyl chains in the lipid membrane interact with each other by the van der Waals–London forces [53]. When the interaction among fatty acyl tails increases, the C–H stretching peak is shifted toward the lower wavenumbers, indicating hardening of the membrane, i.e., a decrease in the cancer cell membrane fluidity. Additionally, the calculated ν_s_(CH_2_)/ν_s_(CH_3_) area ratios revealed statistically significant increased lipid chain packing after all treatments compared to control (Figure 6).

**Figure 7:**
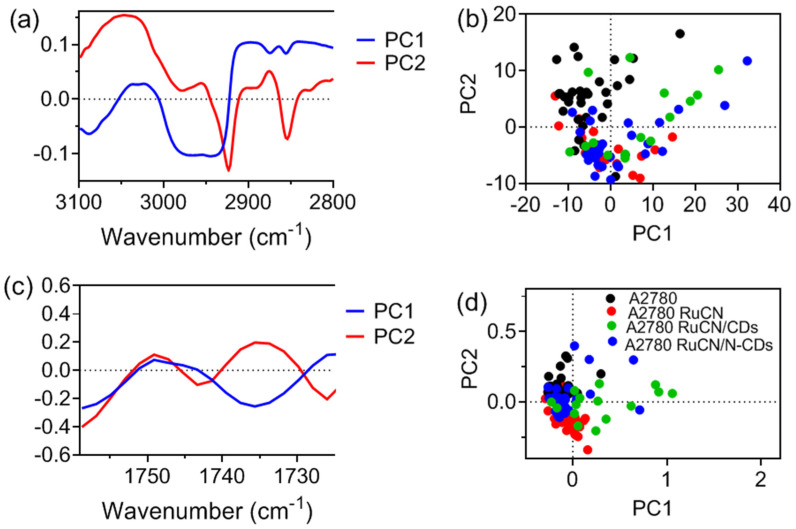
PCA loadings of the first two components (PC1 and PC2) of ν(C–H) vibration region (**a**) and corresponding PCA score plot (**b**). PCA loadings of the first two components (PC1 and PC2) of ν(C=O) vibration region of lipids (**c**), and corresponding PCA score plot denote the variability associated with the first two components (**d**).

PCA was performed to observe significant differences between the spectra of control and treated groups and assign the changes more precisely. PC loadings for cells in the ν(C–H) region (Figure 7a) show maximum differences in the bands at 2925 and 2850 cm^−1^, which correspond to ν_as_(CH_2_) and ν_s_(CH_2_), respectively. Thus, maximal pronounced spectral changes indicate alternation of lipid structure in the methylene groups. Since lipid acyl chains are orientated within the cell membrane and the CH_3_ groups are located in the hydrophobic interior, the vulnerability of CH_2_ groups is higher than that of CH_3_ groups. PC loadings in the ν(C=O) region (Figure 7c) show maximum differences in the signal at 1735 and 1742 cm^−1^.

PCA scores of ν(C–H) and ν(C=O) in the lipid region (Figure 7b,d) show that untreated A2780 cells (control, black dots) and cells treated with RuCN (red dots) are clearly differentiated from other groups of treated cells. While the PC2 scores for untreated A2780 cells show mostly positive values, the PC2 scores for cells treated with RuCN show negative values. This points toward the most prominent change in membrane lipids after the treatment with RuCN. On the contrary, after the treatment with RuCN/(N-)CDs, PC1 and PC2 values are localized in the positive and negative values in the PC1 × PC2 graph (Figure 7b,d).

#### 3.3.3. Impact of Various Anticancer Treatments on the Membrane Surface Hydration

**Figure 8:**
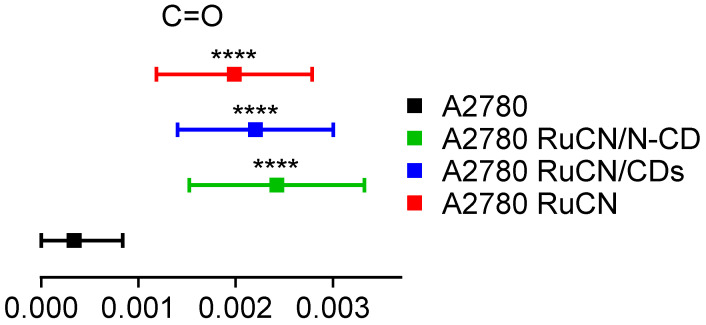
The changes of the C=O band peak area after the indicated treatment conditions. The values are presented with the mean ± SD, and all the values are compared to the control (A2780 untreated cells, black color). **** indicates values that are significantly different (*p* ≤ 0.0001).

Treatment with RuCN and RuCN/(N-)CDs could influence the lipid membrane structure, as well as the hydration water structure at the membrane–water interface. The ester carbonyl groups in the cell membrane bilayer are part of the hydrophilic head polar groups that are in contact with the aqueous environment and can form hydrogen bonds with surrounding water molecules. FTIR can estimate the level of hydration or dehydration of the membrane surface using the carbonyl band behavior. Because of that, the band’s frequency and relative intensity are sensitive to changes of polarity in the local environment and influenced by hydrogen bonding and interactions with different ligands [49]. Statistically significant peak area changes in the ν(C=O) region were detected after all treatment conditions (Figure 8).

Figure 7c shows that maximal pronounced spectral changes in the ν(C=O) region are detected at 1742 and 1735 cm^−1^. According to the literature, a single C=O band (1760–1725 cm^−1^)in the IR spectrum represents the superposition of two underlying components due to two different forms of carbonyl–water interactions [54]. Therefore, changes in these high- (1742 cm^−1^) and low-frequency (1735 cm^−1^) components can be attributable to free and hydrogen-bonded ester carbonyl groups, respectively. A nearly symmetric ν(C=O) stretching band is centered at 1742 cm^−1^ in untreated A2780 cells (Figure 4b), indicating that the lipid carbonyl is in a relatively unhydrated state, whereas after all treatments, the contours of the ester carbonyl band are fairly broad and shifted toward lower frequencies. Signal broadening and decrease in frequencies denote the higher hydration state of the C=O group after treatments compared to untreated control.

**Figure 9:**
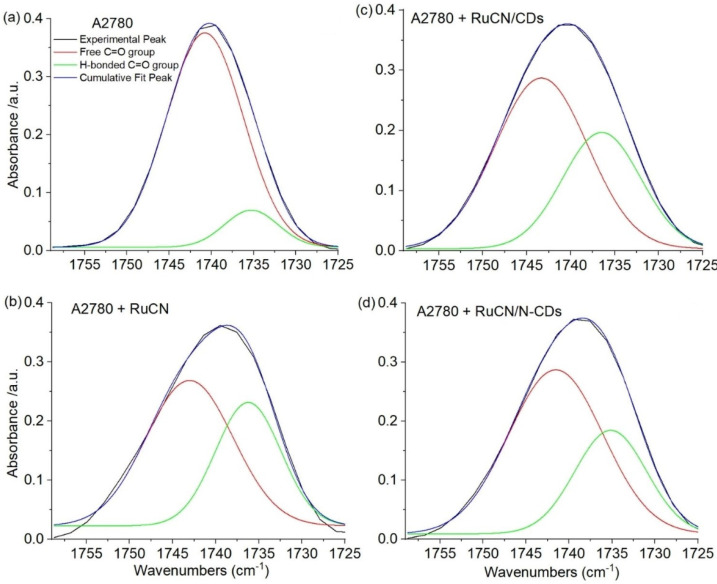
Deconvolved spectrum of C=O vibration band region (1725–1760 cm^−1^) of untreated cells (**a**) after the treatment with RuCN (**b**), RuCN/CDs (**c**), and RuCN/N-CDs (**d**). The black curve shows the original experimental peak, whereas the purple line represents the cumulative fit peak, i.e., the sum of the subcomponent contributions (free C=O group (red line) and H-bonded C=O group (green line)).

In accordance with the literature data, we analyzed the C=O vibrational bands by deconvolution based on the Gaussian function to visualize two subcomponents. The change in relative intensities of the subcomponents revealed distinct differences in the extent of hydrogen bonding to the ester carbonyls depending on the treatment used, as can be seen from Figure 9a–d.

The fact that the intensity of the H-bonded (green) signal increases is consistent with the interpretation that there is interconversion from “free” to hydrogen-bonded carbonyl moieties after treatments. In order to better understand the changes induced by different treatments, the percentages of C=O groups containing H-bonds relative to the total number of these groups were calculated as already described in the literature [55] by using the following equation:

(4) $$\%\;\mathrm{of}\;H-\mathrm{bonded}\;C=O\;\mathrm{groups}=\frac{I\;H-\mathrm{bonded}\;C=O}{I\;H-\mathrm{bonded}\;C=O+I\;\mathrm{free}\;C=O}\times 100$$

where I H-bonded C=O corresponds to the intensity of the H-bonded carbonyl group (1735 cm^−1^) and I free C=O corresponds to the intensity of the unbonded carbonyl group (1742 cm^−1^).

The calculated percentages of C=O groups containing H-bonds relative to the total number of these groups in control and treated cells are given in Table 4. After all treatments, the interconversion from “free” to hydrogen-bonded carbonyl moieties was detected. At least two possibilities can explain this observation. One is that RuCN and RuCN/(N-)CDs bind to nonhydrated C=O groups. The other is that the carbonyl groups become more exposed to water after changes induced by RuCN and RuCN/(N-)CDs due to the changed packing of the fatty acyl chains in the lipid bilayer, as shown in Figure 6. Although the increase in hydration water structure at the membrane–water interface was detected after all treatment conditions compared to untreated control (Figure 9, Table 4), the effect was most pronounced with RuCN, just like in oxidative stress mediating (Figure 5b) and change in lipid chain packing ν_s_(CH_2_)/ν_s_(CH_3_) (Figure 6). These results are in accordance with the fact that the drug’s hydrophobic nature, size, and charge play a crucial role in drug–lipid interactions.

## 4. Conclusions

We have demonstrated that the RuCN complex induces significant changes in the lipid moiety of A2780 ovarian cancer cells. These changes are related to the increased extent of the lipid peroxidation (and production of lysophospholipids) and changes in the phospholipid arrangement or membrane fluidity. Nanocarriers, N-CDs, revert the peroxidation process but do not significantly affect RuCN-induced changes in the lipid bilayer rearrangements. This is documented by the percentage of the hydrogen-bonded C=O groups and lipid chain packing, which substantially increase in the treatment with RuCN and remain similar in the presence of (N-)CDs. On the other hand, RuCN/(N-)CDs exhibit similar effects on cell cytotoxicity.

Minimizing the oxidative stress induced by a metallodrug is the desired property in tumor therapy because the extent of necrotic tissue is lower. Taken together, we have demonstrated that the addition of N-CDs to the tumor therapeutic system suppresses the level of oxidative stress, still keeping the antitumor activity and the potential to penetrate through the lipid bilayer and reach the target molecules. These effects are related to the physicochemical properties of the RuCN/(N-)CDs system and its components. However, further studies are required on additional ovarian cancer cell subtypes to elucidate better the mechanism of action and the effect on other biomolecular species.

## Figures and Tables

**Table 1:** Molecular representation and physicochemical properties (zeta potential, mean size, and partition coefficients) of nanocarriers CDs and N-CDs.

	Structural Formula	Electrical Charge Zeta Potential	Mean Size	Lipophilicity log K_o/w_
**Ru Complex**	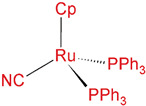 Ph-C_6_H_5_, Cp-η^5^-C_5_H_5_	neutral	μm-sized	>+3 *
**CDs**	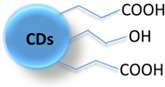	−6.6 mV	7–8 nm	−0.84
**N-CDs**	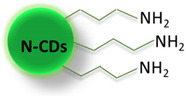	7.8 mV	7.8 nm	−1.73

* According to ACD/ChemSketch, the contribution of individual lipophilicity of ligands PPh3, Cp, and CN to the total lipophilicity of complex is 5.69 ± 0.20, 1.79 ± 0.21, and −0.25 ± 0.19, respectively. Software (version 12.0) predicts accurate partition coefficient values from the chemical structures.

**Table 2:** Signal position and the assignment of cation species (^+^) related to lipids obtained from mass spectra. Peaks with an asterisk (*) correspond to the higher molecular weight oligomers of the DHB matrix.

Signal Position (*m*/*z*)	Signal Assignment	Signal Position (*m*/*z*)	Signal Assignment
449.4	[LPA (16:0) + 2H + K]^+^	686.9	[PA (16:0; 16:0) + K]^+^
483.3	[LPA (18:0) + H + 2Na]^+^	715	[PE (16:0; 18:2) + H]^+^
498.3	[LPE (16:0) – H + 2Na]^+^	735.5	[PA (16:0; 20:4) + 2H + K]^+^
501.2	[LPE (18:0) + Na]^+^	760.6	[PC (16:0; 18:1) + H]^+^
504.3	[LPE (20:4) + H]^+^	767.5	[PG (16:0; 16:0) + 2Na]^+^
538.9	nonidentified	773.5	[PG (16:0; 18:0) + H + Na]+
544.3	[LPC (18:1) + Na]^+^	782.6	[PC (16:0; 18:1) + Na]^+^ or [PC (16:0; 20:4) + H]^+^
551 *	Matrix-oligomer	786.6	[PC (18:0; 18:2) + H]^+^
567 *	Matrix-oligomer	808.6	[PC (18:1; 20:4) + H]^+^ or [PC (18:2; 18:2) + H]^+^
570.4	[LPC (22:5) + H]^+^ or [LPS (18:0) + 2Na]^+^	881.4	([PI (16:0; 20:4) + Na]^+^)
590–650	Fragmentation product of PE	953.6	([PI (43:6) + H]^+^) or [PI (21:0; 22:6) + H]^+^
592.3	[LPC (22:5) + Na]^+^ or [LPS (18:0) − H + 3Na]^+^	979.7	[PI (44:0) + H]^+^ or [PI (22:0; 22:0)) + H]^+^

**Table 3:** Band positions and assignments in the SR-FTIR spectroscopy spectra (ν = stretching vibration; s = symmetric; as = asymmetric).

Band Assignment	Band Position (cm^−1^)
ν(C=O)	1760–1725
ν_s_(CH_2_)	2862–2842
ν_s_(CH_3_)	2882–2862
ν_as_(CH_2_)	2945–2900
ν_as_(CH_3_)	2980–2945
ν(C=C–H)	3100–3000

**Table 4:** Percentages of carbonyl groups (C=O) containing hydrogen bonds in untreated and treated A2780 cells. The percentages are calculated on the basis of spectra given in Figure 9a–d.

	Percentage of H-Bonded C=O Groups
A2780	15.69%
A2780 + RuCN	46.28%
A2780 + RuCN/CDs	40.68%
A2780 + RuCN/N-CDs	39.14%

## Data Availability

The data presented in this study are available in this article and supplementary material.

## Supplementary Materials

The following supporting information can be downloaded at: https://www.mdpi.com/article/10.3390/cancers14051182/s1, Figure S1: Cell metabolic activity after the indicated treatment condition with RuCN expressed as a percentage of the nontreated control (the mean±SD). Statistical significance was determined using the ANOVA test. ns, not significant, *p* > 0.05; *, *p* < 0.05; **, *p* < 0.01; ****, *p* < 0.0001; Figure S2: Visible microscopy of (a) untreated A2780 cells (control), (b) A2780 cells treated with RuCN/N-CDs, (c) A2780 cells treated with RuCN/CDs, and (d) A2780 cells treated with RuCN.
