# Supplementary material for: Lipid Status of A2780 Ovarian Cancer Cells after Treatment with Ruthenium Complex Modified with Carbon Dot Nanocarriers: A Multimodal SR-FTIR Spectroscopy and MALDI TOF Mass Spectrometry Study
Source: Cancers (Basel). 2022 Feb 24;14(5):1182. doi: 10.3390/cancers14051182 (PMC8909423; doi:10.3390/cancers14051182)
Supplement: Supplementary file 1 [file cancers-14-01182-s001.zip › cancers-1559733-supplementary.pdf]

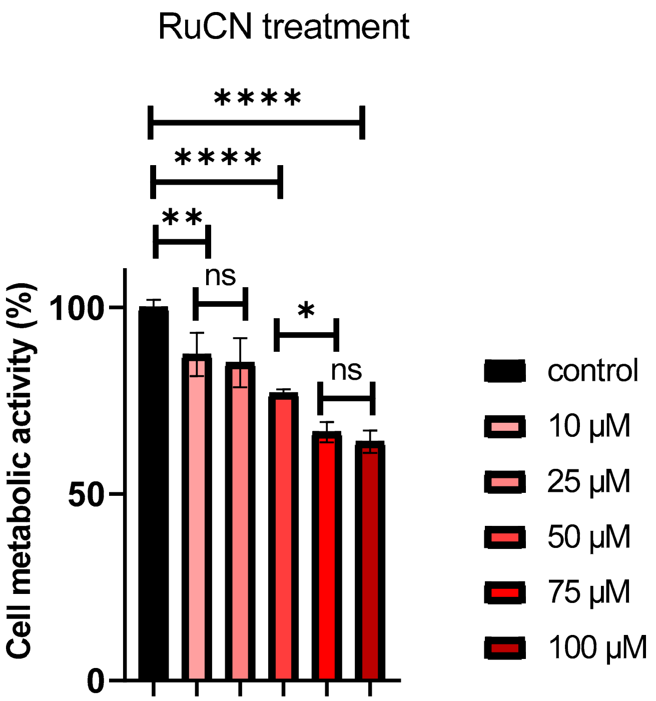

Figure S1. Cell metabolic activity after the indicated treatment condition with RuCN expressed as a percentage of the non-treated control (the mean ± SD). Statistical significance was determined using the ANOVA test. ns (not significant, $p>0.05$) and significant * ($p < 0.05$), **($p < 0.01$), **** ($p < 0.0001$).

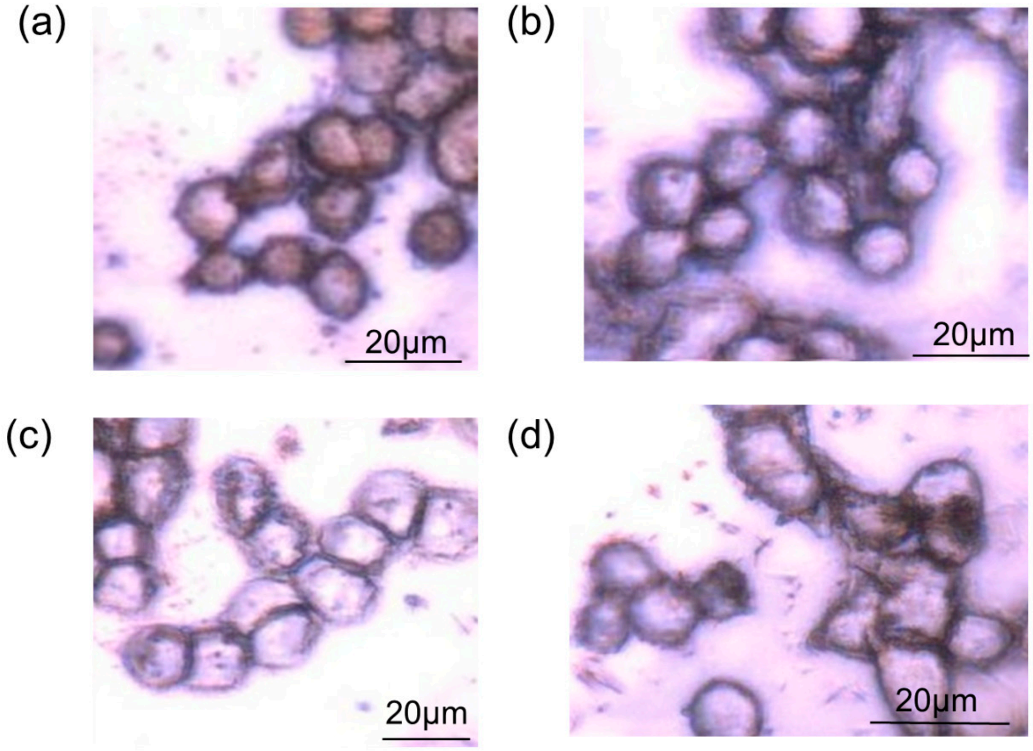

Figure S2. Visible microscopy of (a) untreated A2780 cells (control), (b) A2780 cells treated with RuCN/N-CDs, (c) A2780 cells treated with RuCN/CDs, and (d) A2780 cells treated with RuCN
